# Stem cells and COVID-19: are the human amniotic cells a new hope for therapies against the SARS-CoV-2 virus?

**DOI:** 10.1186/s13287-021-02216-w

**Published:** 2021-03-01

**Authors:** Rodrigo N. Riedel, Antonio Pérez-Pérez, Víctor Sánchez-Margalet, Cecilia L. Varone, Julieta L. Maymó

**Affiliations:** 1 Instituto de Química Biológica (IQUIBICEN), CONICET- Departamento de Química Biológica, Facultad de Ciencias Exactas y Naturales, Universidad de Buenos Aires, Ciudad Universitaria Pabellón 2, 4° piso, 1428 Buenos Aires, Argentina; 2 Departamento de Bioquímica Médica y Biología Molecular e Inmunología, Hospital Universitario Virgen Macarena, Facultad de Medicina, Universidad de Sevilla, Avenida Sánchez Pizjuán 4, 41009 Sevilla, España

**Keywords:** COVID-19, SARS-CoV-2, Stem cells, Stem cell therapy, Mesenchymal stem cells, Amnion, Human amniotic epithelial cells, Human amniotic mesenchymal stromal cells

## Abstract

A new coronavirus respiratory disease (COVID-19) caused by the SARS-CoV-2 virus, surprised the entire world, producing social, economic, and health problems. The COVID-19 triggers a lung infection with a multiple proinflammatory cytokine storm in severe patients. Without effective and safe treatments, COVID-19 has killed thousands of people, becoming a pandemic. Stem cells have been suggested as a therapy for lung-related diseases. In particular, mesenchymal stem cells (MSCs) have been successfully tested in some clinical trials in patients with COVID-19. The encouraging results positioned MSCs as a possible cell therapy for COVID-19. The amniotic membrane from the human placenta at term is a valuable stem cell source, including human amniotic epithelial cells (hAECs) and human mesenchymal stromal cells (hAMSCs). Interestingly, amnion cells have immunoregulatory, regenerative, and anti-inflammatory properties. Moreover, hAECs and hAMSCs have been used both in preclinical studies and in clinical trials against respiratory diseases. They have reduced the inflammatory response and restored the pulmonary tissue architecture in lung injury in vivo models. Here, we review the existing data about the stem cells use for COVID-19 treatment, including the ongoing clinical trials. We also consider the non-cellular therapies that are being applied. Finally, we discuss the human amniotic membrane cells use in patients who suffer from immune/inflammatory lung diseases and hypothesize their possible use as a successful treatment against COVID-19.

## Introduction

The COVID-19 (coronavirus disease-2019) is a new respiratory disease caused by the SARS-CoV-2 (severe acute respiratory syndrome coronavirus 2) virus. This virus was identified in several pneumonia patients in late December 2019 in Wuhan city, Hubei province, in China. Since then, the COVID-19 contagion has occurred exponentially worldwide, becoming the most urgent problem of global public health [1, 2].

Such was the level of spread and severity of the disease that on March 11, 2020, the WHO (World Health Organization) decided to declare it as a pandemic disease [2]. By December 21, 2020, approximately 77 million people were infected and more than 1.6 million deaths from COVID-19 have been reported. The global mortality due to COVID-19 oscillates between 2 and 10% approximately.

The COVID-19 is characterized by a lung infection that in several cases can trigger a multiple proinflammatory cytokine storm. This cytokine storm causes different lung traumas including air exchange dysfunction, edema, acute respiratory distress, and secondary infection. Moreover, pre-existing comorbidities, older age, and male sex are the major risk factors for complications. These conditions could result in death, without effective treatment [3, 4].

Since its appearance, different biomedicine experts from all over the world mobilized to discover a treatment to control this pandemic disease. Therefore, the recent outbreak of COVID-19 highlights the urgent need for safe and effective therapies, especially for the most severe cases.

Stem cells have always been a focus of attention for regenerative medicine and cellular therapies. Mesenchymal stem cells (MSCs) in particular have low immunogenicity, appreciable differentiation capability, and outstanding immunomodulatory properties [5]. Moreover, MSCs can oppose viral infection because of their enhanced cytokine quality production. MSCs are present in almost all tissues, and their properties and characteristics are different depending on the tissue. Properties of stem cells isolated from the human placenta are unique since they are easy to obtain without invasive procedures and have no ethics concerns. In addition, stem cells from the amniotic membrane are obtained in large quantities, they are not tumorigenic, and they can be used in allogenic transplants [6]. Based not only on their strong immunomodulatory properties but also due they mainly accumulate in the lung, experts began to consider MSCs as a promising source of COVID-19 therapy. In fact, many clinical trials have been elicited.

Here, we review and discuss the general characteristics of COVID-19, the current clinical trials with MSCs, and the possible therapeutic options using the human amniotic membrane stem cells.

## Coronavirus and COVID-19: characteristics and mechanisms of infection

Viruses are acellular infectious particles containing either RNA or DNA (single- or double-stranded) that can infect all life forms.

Coronaviruses (CoVs) are a group of enveloped viruses with a non-segmented positive-sense RNA genome, of approximately 30 kb. The CoVs have a spherical shape with a solar corona appearance. This appearance results from the presence of a lipid envelope studded with spike projections that emanate from the virus surface [7].

**Fig. 1:**
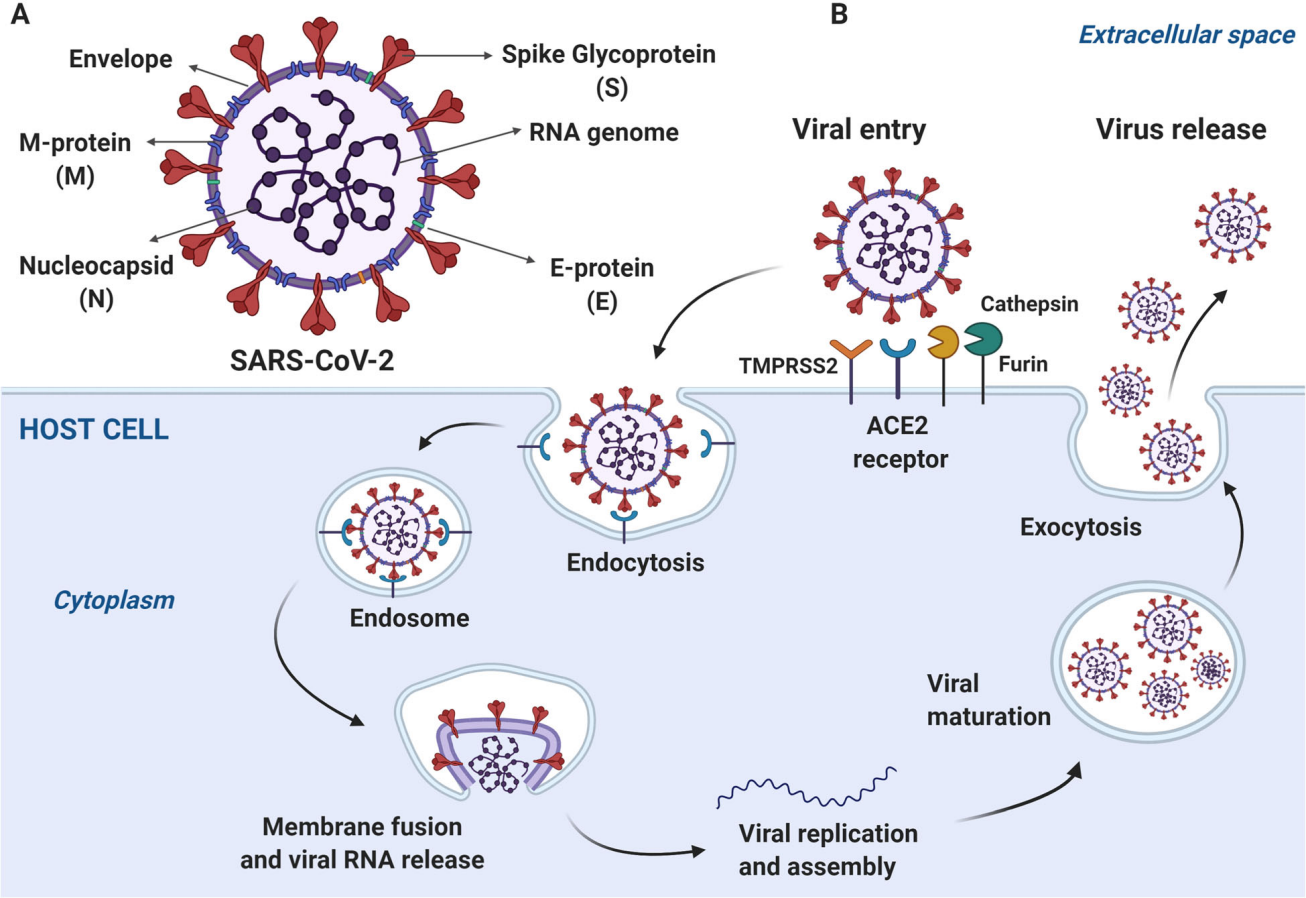
SARS-CoV-2 structure and cell entry. **a** The coronavirus (CoVs) structure is complex and includes the three principal structural proteins: the spike glycoprotein (S), the transmembrane glycoprotein (M), and the nucleocapsid protein (N). The glycoprotein S is found in the viral envelope. The envelope (E) protein is a minor transmembrane protein present in the structural region. The CoVs genome is formed by a positive sense single-stranded RNA of 30 kb in size. **b** As a coronavirus, SARS-CoV-2 uses the S glycoprotein to facilitate entry to the host cell. This virus principally targets the respiratory epithelial cells, that express the angiotensin-converting enzyme 2 receptor (ACE2). The S protein binds to ACE2 allowing the virus access to the host cells cytosol. Proteases like TMPRRS2, cathepsin, or furin, help to viral and cellular membranes fusion through the S protein cleavage. After the viral entry, the replicase gene from the virion genomic RNA is translated. Then, the viral RNA is synthesized and the replicase complexes assembly. In the endoplasmic reticulum occurs the translation of the structural glycoproteins S, M, and E. All the translated proteins interact for CoVs assembling. Once the virions are formed, they are transported to the host cell surface in vesicles and then released by exocytosis. (This figure was created with BioRender.com)

The SARS-CoV-2 virion measures 60–140 nm with club-shaped glycoprotein spikes on the surface and 10 ORFs. These ORFs encode the complete viral genome, including the replicase polyprotein 1ab and the viral structural proteins (membrane, envelope, spike, nucleocapsid and auxiliary proteins) [8] (Fig. 1a).

The SARS-CoV-2 virus data would indicate that the most likely primary source of COVID-19 would be the bats [9]. However, it is not known yet whether there is an intermediate host between bats and humans [10].

The contagion rate and the transmissibility are very high. Most of the evidence proposes that the transmission of SARS-CoV-2 between humans occurs when an infected person coughs, sneezes, talks, or sings [11].

The host infection SARS-CoV-2 mechanism consists in the spike glycoprotein (S) binding to receptor 2 of the angiotensin-converting enzyme I (ACE2) [12]. This receptor is expressed in almost all organs of the body, especially in alveolar type II cells that produce surfactant factor [13–15]. The SARS-CoV-2 also enters the cells via a pH-dependent endocytosis, mechanism, and involving the proteases accessibility in the host. Cathepsins, furin, TMPRSS2, and human airway trypsin-like protease (HAT) control CoVs entrance to the cells through the plasma membrane or endocytosis. The cellular serine protease TMPRSS2 is required to correctly process the SARS-CoV-2 spike protein and facilitate the host cell entry [15] (Fig. 1b).

## Pathology development

Once hCoV invades host cells, it activates the innate immune system. The immunity system triggers a release of inflammatory factors that limits the expansion of the virus and stimulates its phagocytosis by macrophages. However, SARS-CoV-2 can evade the innate immune response, mainly by increasing the number of copies in the airway epithelium [16]. Then, the adaptive immune response, mediated by B and T lymphocytes, activates the production of hCoV-specific antibodies, and the release of proinflammatory cytokines, respectively [17, 18]. The regulation of the antibody response in patients infected with SARS-CoV-2 is largely unknown.

It is estimated that 81% of patients infected with SARS-CoV-2 suffer from mild or asymptomatic disease. The most common symptoms in these cases are fever, fatigue, dry cough, and shortness of breath. On the other hand, 14% of patients manifest severe conditions. Among these cases, it is calculated that 20% are patients with diabetes, 15% with hypertension, and 15% with other cardiovascular diseases. All severe patients develop pneumonia [19]. In general, this pneumonia is evidenced by bilateral patchy shadows or ground-glass opacity (GGO) in computed tomography (CT) lung scans. The rest of the patients suffers from a serious illness featured by severe pneumonia with respiratory insufficiency. This respiratory condition triggers in a short period the acute respiratory distress syndrome (ARDS), septic shock, and multiorgan dysfunction [20, 21].

The ARDS is characterized by an overreaction of the immune system. The result is a massive inflammatory cell infiltration and the release of pro-inflammatory cytokines and chemokines including interleukin (IL)-6, IL-8, IL-1β, interferon-gamma (IFN-γ), granulocyte colony-stimulating factor (GSCF), monocyte chemo-attractant protein 1 (MCP1), macrophage inflammatory protein 1 alpha and 1 beta (MIP1A, MIP1B), tumor necrosis factor-alpha (TNF-α), CCL-2, CCL-3, CCL-5, and interferon γ inducible protein (IP-10) [19, 22]. These proteins attract macrophages, monocytes, and T cells to the infection site promoting additional inflammation and beginning a pro-inflammatory feedback loop. Thereby, the malfunctioning immune response conduces to further accumulation of immune cells in the lungs. This accumulation triggers a pro-inflammatory cytokine overproduction which eventually damages the lung structure [23]. Like in other coronavirus infections, during COVID-19 there is an attempt to repair and remodeling the lungs by fibroproliferation. This derives in a potential risk of developing pulmonary fibrosis, which in fact may occur as a consequence of SARS-CoV-2 infection. Fibrotic sequelae are found in CT lung scans in COVID-19 patients [24].

The origin of the cytokine storm triggered by the coronavirus occurs in the infected area and rapidly expands through the central body circulation. This cytokine storm spreads throughout the body, causing the decline of patient condition and producing, in most cases, its death due to multiorgan failure [25].

**Fig. 2:**
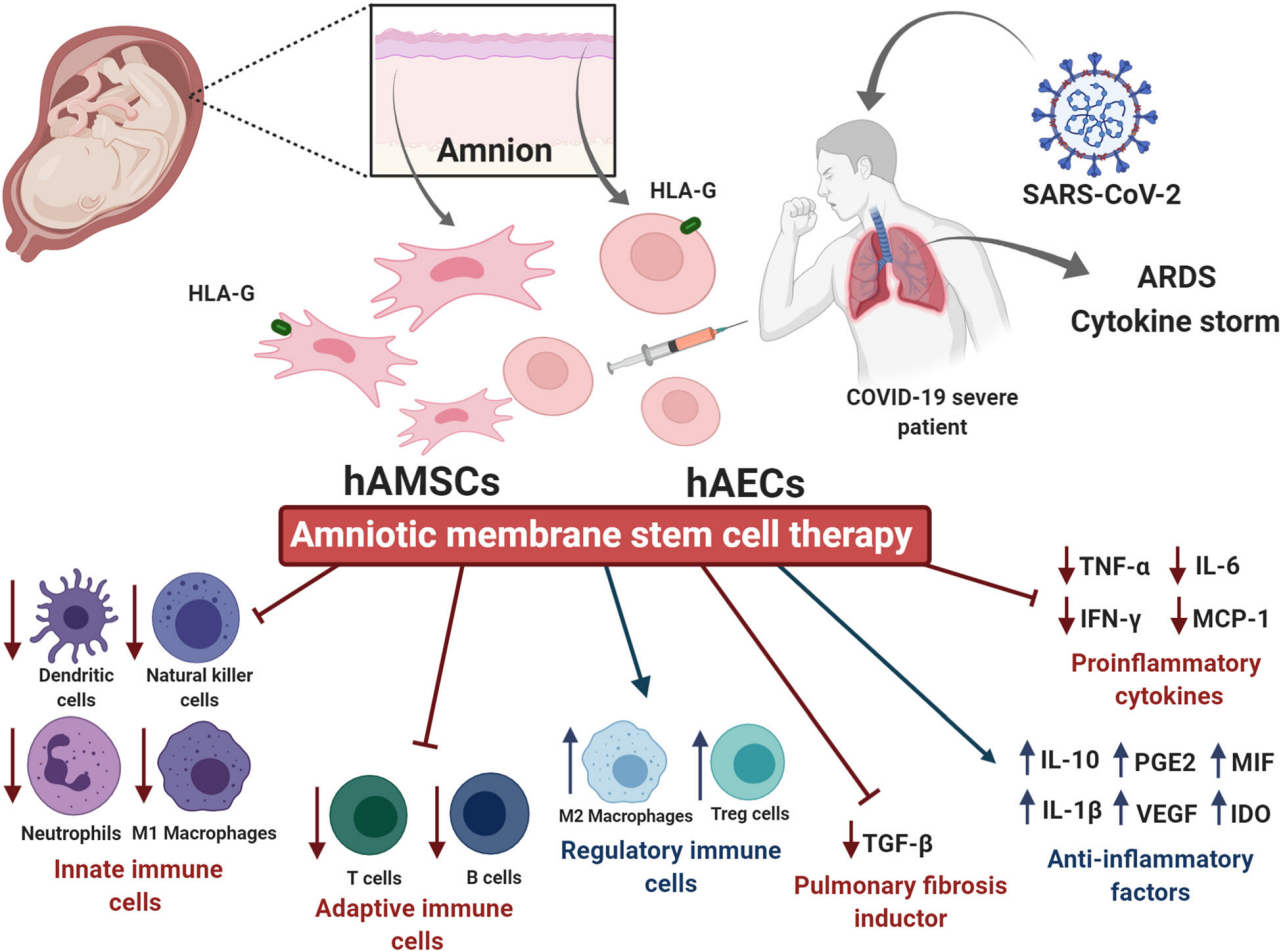
Perspectives of amniotic membrane stem cell therapy against COVID-19. Stem cells isolated from the amnion of the human placenta at term include hAECs and hAMSCs. HAMSCs and hAECs have immunoregulatory, anti-inflammatory, and regenerative properties. These features position amniotic stem cells as candidate for a successful treatment against COVID-19. The amniotic membrane stem cell therapy could attenuate the cytokine storm that occurs in acute respiratory distress syndrome (ARDS) caused by the SARS-CoV-2 virus. Amnion cells could modulate the proliferation, function, and migration of immune cells such as T and B lymphocytes, NK cells, DC, neutrophils, and macrophages. Thus, hAECs and hAMSCs would stimulate regulatory immune cells decreasing the pulmonary inflammatory microenvironment. Moreover, amniotic membrane cells would reduce proinflammatory cytokines (TNF-α, IFN-γ, IL-6, and MCP-1) and increase the release of soluble anti-inflammatory factors (IL-10, IL-1β, PGE2, MIF, VEGF, and IDO). Additionally, amnion cells could exert an anti-fibrotic effect at the lung injury focus through TGF-β factor inhibition. Altogether, human amniotic membrane stem cell therapy would prevent multiple organ failure progression in patients who suffer from COVID-19 (This figure was created with BioRender.com)

Severe affected people age goes from 60 years and over probably due to a dysfunctional immune response development and/or a failure in virus elimination [26]. On the other hand, most of the children younger than 18 years are asymptomatic or develop mild symptoms [27]. In this context, the set of immune factors that lead to a severe inflammatory response are still unclear, and consequently possible treatments are also uncertain (Fig. 2).

## Stem cell therapies for COVID-19

Stem cell contribution to current medicine is central, both for their use in basic research, and for the opportunities to develop innovative therapies in clinical practice. The urgency of finding new and effective treatments against COVID-19 has positioned stem cells at a strategic place in the scene. Since COVID-19 emerged, several groups have dedicated their efforts to this topic. In this way, Han et al. [28] have derived lung organoids from human-induced pluripotent stem cells in order to analyze COVID-19 consequences and possible drug treatments. The alveolar type II cells from the organoids expressed ACE2 and were susceptible to virus infection. They also tested the potential of virus infection in mouse xenografts models. In both cases, the presence of SARS-CoV-2 virus was detected after 24 h of infection. This group identified some drug candidates—approved by the Food and Drug Administration (FDA)—that were able to block the RNA virus replication: imatinib, mycophenolic acid, quinacrine dihydrochloride, chloroquine, and prochlorperazine. They provided evidence that support the use of these drugs in clinical trials.

In addition, as the adipose stem cells (ASC) and the stromal vascular fraction (SVF) possess a high secretory activity of proangiogenic factors and immunomodulatory properties, they have been considered for COVID-19 patient treatment. Indeed, both ASC and SVF, as well as microRNAs derived from ASC, could be injected in these patients in a quick and safe way [29].

Human embryonic stem cells (hESC) have also been studied as a possible COVID-19 treatment, for lung fibrosis condition [30]. In this study, authors demonstrated that MSCs like derived from hESC are capable to modulate and regulate extracellular matrix production in a bleomycin-induced model of lung injury. These cells also displayed strong immunomodulatory and antifibrotic functions, being able to attenuate lung injuries in vivo. Additionally, their transplantation both in mice and monkeys have shown an excellent safety profile.

MSCs are the most widely used stem cells in medical therapies. MSCs are multipotent cells that can be isolated from the bone marrow, adipose tissue, umbilical cord, dental pulp, and placenta. These cells have immunomodulatory properties that not only protect organs and tissues from a variety of lesions but also favor their regeneration. Besides, such properties allow both allogeneic and autologous use. Most of MSCs are obtained in low number, so in vitro expansion is necessary. However, they have a high proliferation rate which ensures enough cell quantity for clinical application [9]. Moreover, they release anti-inflammatory, antibacterial, antiviral, and immunoregulatory factors, locally in the lesion site [31, 32]. MSC applications in medicine are quite safe [33]. Recently, some reviews works have highlighted the capacity of MSCs to modulate inflammatory response and consequently to protect from COVID-19 lung injuries [34, 35].

In 2004, the first published report about the clinical use of MSCs for a pediatric patient with acute refractory graft-vs-host-disease (GVHD) appeared [36]. Since then, data relative to MSC immunoregulatory properties have raised, until reaching approximately one third of current trials. Most of them are based on CD4 T-lymphocytes, dendritic cells (DC), and natural killer (NK) cell activity modulation [37]. After MSCs intravenous delivery, 80–90% of cells migrate and stay in the lungs. Curiously, most clinical trials studying MSC effect on lung diseases were scarce until this year. Nowadays, promising results are being obtained from preclinical studies and clinical trials related to several diseases characterized by predominant inflammation such as ARDS [38–41].

One interesting approach to treat COVID-19 is the use of exosomes derived from MSCs. Exosomes contain several chemokines, growth factors, microRNAs, and mRNA that can exert paracrine and endocrine anti-inflammatory, anti-fibrotic, regenerative, and immunomodulatory effects [42]. Thus, some authors propose exosomes derived from bone marrow mesenchymal stem cells (BM-MSC) as a new therapy option for COVID-19. In their study, Sengupta et al. [43] treated 24 COVID-19 patients with exosomes derived from BM-MSC, and after 14 days, they observed a survival rate of 83%, without adverse events. In recovered patients, oxygenation was restored and cytokine storm was inhibited.

Pathologies originated by SARS-CoV-2 in organs and tissues are multiple and not only limited to respiratory tract. The immune system exacerbation, cytokine storm, and pulmonary damage and fibrosis could lead to ARDS, with a secondary risk of bacterial infection. In addition, other organs like the liver, heart, brain, kidneys, or gut could also be affected by COVID-19. In this context, and given the pleiotropic activities that could exert in this disease, MSCs should be considered as a potential treatment. The beneficial actions of MSC consist not only of immunomodulation and anti-inflammation properties, but also in promoting tissue regeneration and repair (Table 1). MSC transplantation can reduce the immune system exacerbation by inhibition of the innate immune response, the release of immunomodulatory molecules, and the inhibition of the adaptive immune cell proliferation and activation [44, 45]. Cytokine storm could be ameliorated after the release of anti-inflammatory molecules and increase of Treg cells, Th22 cells, and M2 macrophages phenotype [46–48]. Also, MSCs secrete LL-37 peptide that has an antibacterial effect [32]. MSCs inhibit TGF-β1 and collagen deposition in the lungs promoting an antifibrotic effect. Moreover, the tissue damage can be repaired since MSCs induce neovascularization and stem cell differentiation and proliferation [49, 50].

**Table 1:** Pathologies involved in COVID-19 infection and MSC mechanisms to counteract the infection damages

Organs/tissue	SARS-CoV-2 associated pathologies	Mechanisms of MSC action
Respiratory tract	Immune system exacerbation Cytokine storm Secondary infection (microbial invasion) Pulmonary architecture damages Pulmonary fibrosis	*Immunomodulation* -Inhibit innate immune cell activation (NK, DC, neutrophils, macrophages) -Inhibit adaptive immune cell proliferation and activation (T and B cells) -Immunomodulatory molecules release (HGF, IDO, PGE2) *Anti-inflammation* -Anti-inflammatory cytokine release (IL-10, TGF-β) -Increase Treg and Th22 cells -Induce M2 macrophages phenotype *Antibacterial effect* -Release antibacterial peptide (LL-37) *Regeneration* -Induce neovascularization -Stimulate local stem cell differentiation and proliferation -Secrete angiopoietin-1 and FGF-7 -Increase bioenergetic levels through mitochondrial transfer *Fibrosis reduction* -Inhibit TGF-β1 and collagen deposition in the lungs
Heart	Myocarditis Arrhythmia Acute coronary syndrome Coagulopathies Kawasaki syndrome	*Immunomodulation* -Reduce IL-1β, IL-12, IL-17, C–C motif chemokine ligand 5, INF-ϒ, and TNF-α levels -Polarize macrophages towards an anti-inflammatory state *Antiapoptotic effect* -Reduce apoptosis of cardiomyocytes *Fibrosis reduction* *Neoangiogenic effect* -Enhance angiogenesis
Liver	Cholestasis Hepatitis	*Immunomodulation* Immunomodulation of T helper (Th) cells Reduction of TNF-α, cytokeratin 19, metallopeptidase 9 and MCP-1 Improvement of monocyte infiltration in bile ducts
Kidneys	Acute kidney injury (AKI)	Immunomodulation Inhibition of apoptosis Promotion of angiogenesis Nephroprotective effects Regeneration of renal tubular cells through MSC-derived EVs
Digestive tract	Gastrointestinal (GI) alterations Deep alterations in gut microbiota	Inflammation regulation Induce the restoration of composition and diversity of colonic bacteria Promote tissue remodeling Induce eradication of pathogenic bacteria
Central nervous system	Stroke Headache Epileptic seizures Depression Encephalitis Dizziness Guillain-Barre syndrome	Immunomodulation Promote angiogenesis Induce neuroprotection. Re-establishment of neural circuit Help to reduce viral invasion Induce reprogramming of microglia from M1 to M2 phenotype Downregulate TNF-α, IL-1β, and IL-6

*HGF* hepatocyte growth factor, *IDO* indoleamine 2,3-dioxygenase, *PGE2* prostaglandin E2, *TGF-β* transforming growth factor β, *Treg cells* T regulatory cells, *Th22 cells* T helper cells 22, *EVs* extracellular vesicles

Some cardiovascular pathologies like arrhythmias, acute coronary syndrome, myocarditis, coagulopathies, and Kawasaki syndrome were described to be associated with COVID-19 [51–53]. Either due to their antiapoptotic, neoangiogenic, or immunomodulatory effects, MSC has demonstrated to exert protective actions in several cardiovascular diseases [54–56]. Similar effects of MSC and their EVs (extracellular vesicles) have been reported in acute kidney injury models [57]. The liver has also been reported to be injured during COVID-19, mainly by cholestasis and hepatitis [58]. In animal models, MSCs have improved hepatitis B condition [59]. Sclerosing cholangitis was also ameliorated by modulation of Th cells and reduction of pro-inflammatory molecules [60]. COVID-19 patients suffered gastrointestinal alterations, including gut microbiota alteration [61]. MSCs can regulate inflammation and restore composition and diversity of colonic bacteria [62, 63]. The brain is affected by SARS-CoV-2. When the loss of taste and smell was detected, the capacity of the virus to invade and affect the nervous system became in evidence. Actually, diseases associated with SARS-CoV-2 include stroke, headache, epileptic seizures, depression, encephalitis, dizziness, and Guillain-Barre syndrome [64]. Some studies have shown that MSCs are able to improve these conditions mainly by immunomodulation, neuroprotection induction, angiogenesis promotion, and reduction of the proinflammatory environment [65–67].

Currently, there are 41 clinical trials using MSCs as cell therapy to combat SARS-CoV-2 pneumonia. Moreover, 21 of them use MSCs derived from the human placenta (Table 2). Some of them have been completed and data have been published.

**Table 2:** Clinical trials for COVID-19 treatment using human placenta MSCs

Clinical trial ID	Title	Cell type	Status	Phase	Outcome measures	Locations
NCT04390139	Efficacy and safety evaluation of mesenchymal stem cells for the treatment of patients with respiratory distress due to COVID-19	WJ-MSCs	Recruiting	1; 2	-All-cause mortality at day 28. -Safety of WJ-MSC. -Need for treatment with rescue medication. -Ventilator free days. -Duration of hospitalization. -Evolution of markers of immune response. -And 9 more.	Spain
NCT04456361	Use of mesenchymal stem cells in acute respiratory distress syndrome caused by COVID-19	WJ-MSCs	Active, not recruiting	1	-Oxygen saturation. -Oxygen pressure in inspiration. -Ground-glass opacity. -Pneumonia infiltration. -And 4 more.	Mexico
NCT04313322	Treatment of COVID-19 patients using Wharton’s jelly-mesenchymal stem cells	WJ-MSCs	Recruiting	1	-Clinical outcome. -CT Scan. -RT-PCR results.	Jordan
NCT04461925	Treatment of coronavirus COVID-19 pneumonia pathogen SARS-CoV-2) with cryopreserved allogeneic P-MMSCs and UC-MMSCs	P-MMSCs	Recruiting	1; 2	-Changes in length of hospital stay. -Changes in mortality rate. -Evaluation of pneumonia improvement. -Peripheral blood count recovery time. -And 3 more.	Ukraine
NCT04565665	Cord blood-derived mesenchymal stem cells for the treatment of COVID-19 related acute respiratory distress syndrome	CB-MSCs	Recruiting	1	-Incidence of composite serious adverse events. -Overall survival rate. -Determine the treatment effect on clinical parameters, oxygenation and respiratory parameters. -And 10 more.	USA
NCT04494386	Umbilical cord lining stem cells (ULSC) in patients with COVID-19 ARDS	ULSCs	Recruiting	1; 2	-Incidence of dose limiting toxicity. -Changes in levels of blood glucose (mg/dL) from baseline. -Changes in levels of sodium and potassium (mEq/L) from baseline. -And 9 more.	USA
NCT04573270	Mesenchymal stem cells for the treatment of COVID-19	UC-MSCs	Completed	1	-Survival rates. -Contraction rates	USA
NCT04288102	Treatment with human umbilical cord-derived mesenchymal stem cells for severe corona virus disease 2019 (COVID-19)	UC-MSCs	Completed	2	-Change in lesion, ground-glass, or consolidation lesion proportion of full lung volume from baseline to day 10, 28, and 90. -Time to clinical improvement. -And 5 more.	China
NCT04355728	Use of UC-MSCs for COVID-19 patients	UC-MSCs	Completed	1; 2	-Incidence of pre-specified infusion associated adverse events. -Incidence of severe adverse events. -Survival rate after 90 days post first infusion. -Ventilator-free days. -Change in oxygenation index. -And 11 more.	USA
NCT04252118	Mesenchymal stem cell treatment for pneumonia patients infected with COVID-19	UC-MSCs	Recruiting	1	-Size of lesion area by Chest radiograph or CT. -Time of nucleic acid turning negative. -CD4+ and CD8+ T cell count. -And 6 more	China
NCT04416139	Mesenchymal stem cell for acute respiratory distress syndrome due for COVID-19	UC-MSCs	Recruiting	2	-Changes in body temperature. -General biochemical changes in Leukocytes. -Functional respiratory change: PaO2/FiO2 ratio. -And 23 more.	Mexico
NCT04437823	Efficacy of intravenous infusions of stem cells in the treatment of COVID-19 patients	UC-MSCs	Recruiting	2	-Safety and efficacy assessment of infusion associated adverse events. -Chest radiograph or CT scan. -COVID-19 QRT-PCR. -And 3 more.	Pakistan
NCT04333368	Cell therapy using umbilical cord-derived mesenchymal stromal cells in SARS-CoV-2-related ARDS	UC-MSCs	Recruiting	1; 2	-Lung injury score. -Oxygenation index. -In-hospital mortality. -Mortality. -Ventilator-free days. -Cumulative use and duration of sedatives. -And 9 more.	France
NCT04366063	Mesenchymal stem cell therapy for SARS-CoV-2- related acute respiratory distress syndrome	UC-MSCs	Recruiting	2; 3	-Adverse events assessment. -Blood oxygen saturation. -Clinical symptoms. -Respiratory efficacy. -Biomarker concentrations. -Intensive care unit-free days.	Republic of Islamic
NCT04339660	Clinical research of human mesenchymal stem cells in the treatment of COVID-19 Pneumonia	UC-MSCs	Recruiting	1; 2	-The immune function. -Blood oxygen saturation. -Duration of respiratory symptoms. -COVID-19 nucleic acid negative time. -And 4 more.	China
NCT03042143	Repair of acute respiratory distress syndrome by stromal cell administration (REALIST) (COVID-19)	UC-MSCs	Recruiting	1; 2	-Respiratory compliance. -Oxygenation index. -Driving pressure. -Extubation and reintubation. -Ventilation free days at day 28. -And 6 more.	UK
NCT04399889	hCT-MSCs for COVID19 ARDS	UC-MSCs	Recruiting	1; 2	-Survival after 28 days after the first dose of MSCs. -The number of ventilator free days. -And 8 more.	USA
NCT04269525	Umbilical cord (UC)-derived mesenchymal stem cells (MSCs) Treatment for the 2019-novel coronavirus (nCOV) pneumonia	UC-MSCs	Recruiting	2	-Oxygenation index. -28 days mortality. -Hospital stays. 2019-nCoV antibody test. -2019-nCoV nucleic acid test. -Improvement of lung imaging examinations. -And 13 more.	China
NCT04366271	Clinical trial of allogeneic mesenchymal cells from umbilical cord tissue in patients with COVID-19 (MESCEL-COVID19)	UC-MSCs	Recruiting	2	-Mortality from any cause at 28 days. -Patients alive and without mechanical ventilation on day 14. -Patients cured at 15 days. -And 8 more.	Spain
NCT04371601	Safety and effectiveness of mesenchymal stem cells in the treatment of pneumonia of coronavirus disease 2019	UC-MSCs	Active, not recruiting	1	-Changes of oxygenation index. -Detection of TNF-α, IL-10 levels. -Immune cell detection. -And 2 more.	China
ChiCTR2000031430	Clinical study of human umbilical cord mesenchymal stem cells in the treatment of novel coronavirus pneumonia (COVID-19) induced pulmonary fibrosis	UC-MSCs	Recruiting	2	-Coagulation. -High resolution CT for chest. -Blood gas analysis. -Blood routine. -Cytokine analysis. -And 16 more.	China

*MSCs* mesenchymal stromal cells, *WJ-MSCs* Wharton’s Jelly mesenchymal stem cells, *P-MMSCs* placenta-derived multipotent mesenchymal stromal cells, *CB-MSCs* cord blood-derived mesenchymal stem cells, *ULSCs* umbilical cord lining stem cells, *UC-MSCs* umbilical cord mesenchymal stem cells

The first clinical trial reported by Liang et al. [68] evaluated the use of allogeneic human umbilical cord mesenchymal stem cells (hUCMSC) to treat a severe patient with COVID-19. The 65-year-old patient with severe pneumonia, and respiratory and multiorgan failure was intravenously treated with three doses of 5 × 10^7^ hUCMSCs, one dose every 3 days, in combination with antibiotics and α1-timosin. Previously, the patient had received a conventional treatment of antiviral, antibiotics, glucocorticoids, and immunoglobulins that was ineffective. After the second administration, negative serum parameters such as alanine aminotransferase (ALT), aspartate aminotransferase (AST), C-reactive protein (CRP), and bilirubin gradually decreased and other vital signs were stabilized. Also, the number of white blood cells and neutrophils decreased, and the T cells subsets increased, all to their normal levels. CT images showed a significant decrease in the GGO in both the lungs. After 2 days of the third injection, the patient was negative for SARS-CoV-2 and discharged with no side effects. These results suggested that hUCMSC could be used alone or in combination with other immunomodulators as an alternative treatment for severe patients with COVID-19.

The second clinical trial evaluated the therapeutic potential of transplanted hUCMSCs in 7 patients with COVID-19 pneumonia [69]. Among the 7 patients, one presented critical condition, four were in serious condition, and two had mild symptoms. Other three severe patients were included in the study with the administration of placebo as control. All patients had symptoms, including high fever, shortness of breath, and low oxygen saturation. Treatment was based on the administration of a single intravenous infusion of 1 × 10^6^ MSCs per kilogram bodyweight. After 2–4 days of MSC infusion, all patients significantly improved lung function and decreased clinical symptoms. Moreover, peripheral lymphocyte levels increased, accompanied by a shift to a regulatory phenotype of both CD4+ T cells and DC. Pro-inflammatory cytokines secreting immune cells, such as CRCR3 CD4+ T cells, CXCR3 CD8+ T cells, and NK CXCR3 cells decreased from day 3 onwards. The authors also observed a decrease in TNF-α and an increase in IL-10 and vascular endothelial-derived growth factor (VEGF) that correlated with pulmonary regeneration in treated patients. No adverse effects were observed in any of the patients after 14 days of treatment. The authors of this study concluded that MSC intravenous administration significantly improved the inflammatory status of COVID-19 severe patients, in a safe and effective manner. These promising results were probably due to their immunomodulatory, and anti-inflammatory capacity, which ameliorated fibrosis and improved lung function [69].

A similar study [70] reported the recovery of a COVID-19 severe patient after UCMSCs transplantation. This trial was performed in China. The patient improved its situation after 2 days of intravenous cells injection (1 × 10^6^ cells per kilogram of weight). Moreover, 7 days after treatment the patient was discharged. The proinflammatory parameters decreased and regulatory immune cells increased. Thus, UCMSC transplantation was an effective treatment for a critical COVID-19 patient.

Meng et al. [71] also performed a phase I clinical trial with UCMSCs in moderate and severe COVID-19 patients, all of them with pulmonary disease. The trial involved 18 hospitalized Chinese patients, 9 of which received a treatment with three doses of UCMSC intravenous infusion (3 × 10^7^ cells/dose). There were no adverse incidents after UCMSC infusion, except flushing and fever in two patients and transient hypoxia in one treated patient. The UCMSC treatment demonstrated to be safe and well tolerated. However, all patients, treated or not with the stem cells, were discharged after the improvement of clinical and laboratory parameters. A new and bigger phase 2/3 study is being carried out in order to establish the efficacy of treatment to reduce deaths.

New clinical trials with a higher number of SARS-CoV-2-positive patients are needed to validate the safety and efficacy of hUCMSCs. Similar studies are being carried out with other sources of MSCs, like human dental pulp or umbilical cord blood; however, no results have been obtained yet [72–74].

Importantly, as we mention before, iPSC differentiated to alveolar type II cells are susceptible to be infected by SARS-CoV-2, in vitro [28]. The intention of this model was to study the virus biology and to enable drug candidates screening, but it does not appear to be designed for cell therapy against COVID-19. On the other hand, it has been demonstrated that MSCs are resistant to SARS-CoV-2 infection, both in vitro and in vivo. Schäfer et al. [75] have evaluated the possibility that SARS-CoV-2 can infect human ASCs and BM-MSCs. This is an important issue to consider since infection of stem cells could result in a clearance of transplanted cells impeding their therapeutic efficiency. Moreover, MSCs infected by the SARS-CoV-2 could cause undesirable and unknown side effects, including complications, symptoms aggravation, or transformation [76]. They tested one condition without additional stimuli, a second inflammatory condition and a third condition of co-coculture with virus-infected cells (Caco-2 cells). Both BM-MSC and ASC were resistant to virus infection in the three conditions. First, they observed very low expression of ACE2 and TMPRSS2 in MSCs under these conditions. They did not observe virus infection in any of the cases. In addition, MSCs retained their immunomodulation potential. Amniotic fluid MSC yielded comparable results. With a similar objective, Leng et al. [69] performed a clinical trial injecting MSCs in patients with pneumonia caused by COVID-19. They analyzed gene expression of transplanted MSCs by RNA-seq, and they demonstrated that MSCs were negative for ACE2 and TMPRSS2 expression. This would suggest that MSCs are resistant to infection by SARS-CoV-2, which make them a promising candidate to treat COVID-19 patients.

## Human amniotic membrane-derived stem cells as a therapy for respiratory diseases

Advances in regenerative medicine have intensified the search for new sources of stem cells that can be used as a therapy for different pathologies, including respiratory diseases. In recent years, special attention has been paid to the various types of cells that can be isolated from the human fetal membranes, now recognized as an abundant stem cell source with immunoregulatory and regenerative properties [77, 78].

The fetal membranes are composed of three layers: the amnion, the chorion, and the *decidua capsularis* [79]. The amnion is a fetal tissue formed by an epithelial layer, a collagenous basement membrane, and a stroma layer between the chorion and the amniotic sac [80]. Two types of stem cells can be isolated from the human amniotic membrane: the amniotic mesenchymal stromal cells (hAMSCs), distributed in the collagenous stroma, and the amniotic epithelial cells (hAECs), which delimit the amniotic cavity.

Remarkably, hAMSCs and hAECs have unique features that position them as important candidates for cell therapy. These features include their immunomodulatory properties, their differentiation potential, their isolation without ethical questions, their lack of tumorigenicity, and the possibility of both allogeneic and autologous transplants [80–83]. Human amniotic membrane cells can also secrete cytokines with immunoregulatory and anti-inflammatory properties [84], angiogenic factors [85], growth factors associated with cell proliferation and differentiation, and antiapoptotic and antioxidant factors [86].

During many years, the human amniotic membrane has been recognized for its utility in different clinical applications, like damaged tissues reconstruction and skin transplants [87–89]. Interestingly, the amniotic membrane cells possess a variety of properties attractive for clinical use, such as the promotion of re-epithelization, the decrease of inflammation and fibrosis, and the inhibition of angiogenesis and microbial growth [6, 90, 91]. Moreover, the amniotic membrane could be used for cancer therapy because of its antitumoral properties [92–94].

Despite the promising properties of amniotic membrane cells to heal lung diseases, at present, there are no clinical trials registered with this purpose. Here, we describe the most outstanding characteristics of human amniotic membrane stem cells in terms of their ability to improve several lung injuries. Furthermore, these properties open the field for the amniotic membrane cell therapy for pulmonary diseases caused by COVID-19 (Fig. 2).

## Human amniotic mesenchymal stromal cells

Human amniotic mesenchymal stromal cells are multipotent cells that can be obtained from the stromal layer of the human amniotic membrane. Freshly isolated hAMSCs express the classical MSCs markers, such as CD44, CD90, CD73, and CD105 [6]. Some authors have demonstrated the differentiation potential of hAMSCs towards osteogenic, chondrogenic, and adipogenic lineages [95, 96]. Interestingly, hAMSCs display low immunogenicity because of their low human leukocyte antigen (HLA)-ABC expression and the lack of HLA-DR expression [97, 98]. Moreover, hAMSCs possess immunomodulatory, anti-fibrotic, and anti-inflammatory properties, thus allowing their use in cell therapy [99, 100]. Primarily, hAMSCs exert their therapeutic effect by releasing soluble factors [101]. Amnion cells produce immunomodulatory molecules, including IL-10, transforming growth factor β (TGF-β), hepatocyte growth factor (HGF), prostaglandin E2 (PGE2), and indoleamine 2,3-dioxygenase (IDO) that decrease innate and adaptive immune cell activation [102, 103]. In addition, the immunomodulatory effect has also been found by cell-to-cell contact [102, 104, 105].

Many studies reported that hAMSCs inhibit the proliferation and function of T and B lymphocytes, DC, and NK cells. Amnion cells reduced peripheral blood mononuclear cells (PBMC) proliferation of in a dose-dependent manner and suppress the cytotoxic activity of T and NK cells [47, 106]. Magatti et al*.* [107] demonstrated that hAMSCs and their conditioned medium (hAMSC-CM) inhibit B cell proliferation and differentiation by secreting factors such as prostanoids. The same group showed the inhibitory effect of hAMSCs in DC differentiation [108]. Additionally, amnion cells suppress NK cytotoxicity activity against K562 monocyte cells via IL-10 and PGE2 secretion [109]. Moreover, the NK proinflammatory cytokine production (i.e., IFN-γ) was inhibited after coculture with hAMSCs.

The anti-inflammatory effect of hAMSCs includes T helper 1 (Th1) subset polarization to anti-inflammatory Th22 and T regulatory (Treg) cells [47, 110]; in vitro and in vivo inhibition of pro-inflammatory cytokine production, such as IL-1β, IFN-γ, and TNF-α [111, 112]; and reduction of inflammatory M1 macrophages and differentiation towards anti-inflammatory M2 macrophages [109, 113].

Usually, M1 are called classically activated macrophages, and they mainly secrete pro-inflammatory cytokines. M2 are called alternatively activated macrophages, and they mitigate inflammation and promote wound repair [114]. When macrophage response is predominantly M2 type, it is correlated with fibrotic remodeling of organs, especially in the lungs. Macrophages represent around 70% of the immune cells in the lungs (the most abundant immune cells). During pulmonary fibrosis both M1 and M2, macrophages are involved in its pathogenesis and perform airway remodeling. MSC have been found to modulate the macrophage switch from an inflammatory phenotype (M1) to an immunomodulatory phenotype (M2) [115–117]. After this MSC-macrophage interaction, inflammation and tissue repair are usually resolved [99]. By changing macrophage phenotype, MSC induce the secretion of anti-inflammatory cytokines, like IL-10 and reduce the production of proinflammatory cytokines, in early stages of the inflammatory process. In addition, by inhibiting IL-1B and IL-12 secretion, they reduce the Th1 cell activation. MSC also secrete TSG-6 factor that inhibit NF-kB signaling and other proinflammatory pathways. These secretions also are responsible for M1 to M2 polarization. Exosomes secreted by MSC can also regulate macrophage phenotype polarization, promoting tissue healing [118]. As we mentioned before, in severe cases, ARDS condition developed after COVID-19 infection. ARDS usually lead to uncontrolled fibrosis [119]. Macrophage plays a central role in pulmonary fibrosis resolution. Thus, by regulating macrophage polarization and their secreted molecules, MSC is able to promote tissue healing mainly by fibrosis reduction [120].

As MSCs, HAMSCs could be a remarkable resource for lung injury treatment (Table 3) Recently, Li et al. [121] showed that hAMSCs alleviate the inflammatory microenvironment in a neonatal hyperoxic lung injury rat model. The pro-inflammatory cytokine expression including TNF-*α*, IL-6, IL-1*β*, and MCP-1 decreased after hAMSC infusion. Moreover, hAMSCs reduced pulmonary edema. In addition, hAMSC treatment was able to reduce lung fibrosis and inflammation levels in a white smoke inhalation-induced rat model [122]. In this model, the IL-10 levels augmented after hAMSC administration via the tail vein, alleviating the pulmonary inflammation condition. The authors demonstrated that the hAMSC distribution was mainly in the lung tissues. Direct airway delivery of hAMSCs was also investigated [123]. Authors demonstrated that hAMSCs can be successfully atomized in vitro, maintaining the morphology and a high cell viability. This therapy would allow a more uniform spreading of cells throughout the lung, simplifying the administration.

**Table 3:** Preclinical and clinical trials for lung diseases using amniotic membrane cells

Title	Diseases	Cell type	Type of study	Outcome	Cell therapy	Authors
Intratracheal transplantation of amnion-derived mesenchymal stem cells ameliorates hyperoxia-induced neonatal hyperoxic lung injury via aminoacyl-peptide hydrolase.	Neonatal lung injury	CM- hAMSCs EXO-hAMSCs hAMSCs	Preclinical	✓ Reducing pulmonary edema and inflammatory cell infiltration	AI effect	Li et al. [1]
Human amnion-derived mesenchymal stem cells alleviate lung injury induced by white smoke inhalation in rats	Lung injury	hAMSCs	Preclinical	✓ Reducing lung injury, lung fibrosis, CT score, and inflammation levels	AI and AF effects	Cui et al. [2]
Nrf2 transfection enhances the efficacy of human amniotic mesenchymal stem cells to repair lung injury induced by lipopolysaccharide	Lung injury	hAMSCs	Preclinical	✓ Decreasing lung injury, lung fibrosis, and inflammation	AI and AF effects	Zhang et al .[3]
Conditioned medium from amniotic membrane-derived cells prevents lung fibrosis and preserves blood gas exchanges in bleomycin-injured mice-specificity of the effects and insights into possible mechanisms	Lung fibrosis	CM-hAMSCs	Preclinical	✓ Decreasing lung fibrosis and preserving the blood gas parameters ✓ Diminishing lung macrophage levels	IM, AI, and AF effect	Cargnoni et al. [4]
Human amnion epithelial cell transplantation abrogates lung fibrosis and augments repair	Lung fibrosis	hAECs	Preclinical	✓ Reducing inflammation and lung collagen.	AI and AF effects. Differentiation potential.	Moodley et al. [5]
Human amnion epithelial cells repair established lung injury	Lung injury	hAECs	Preclinical	✓ Normalizing lung tissue density, collagen content, and α-SMA production ✓ Diminishing pulmonary leucocytes and fibroblast activation *in vitro*	AI and AF effects	Vosdoganes et al. [6]
Title	Diseases	Cell type	Type of study	Outcome	Cell therapy	Authors
Human amnion epithelial cells mediate lung repair by directly modulating macrophage recruitment and polarization	Lung injury	hAECs	Preclinical	✓ Reducing macrophage infiltration. ✓ Modulating macrophage polarization	IM and AI effects	Tan et al. [7]
Human amnion epithelial cells prevent bleomycin-induced lung injury and preserve lung function	Lung injury	hAECs	Preclinical	✓ Decreasing gene expression of the proinflammatory cytokines ✓ Reducing pulmonary fibrosis and inflammatory cell infiltration	IM, AF and AI effects	Murphy et al. [8]
Human amnion epithelial cells modulate the inflammatory response to ventilation in preterm lambs	Preterm neonatal lung injury	hAECs	Preclinical	✓ Modulating the pulmonary inflammatory ✓ Reducing acute lung injury	IM effect	Melville et al. [9]
Human amnion cells reverse acute and chronic pulmonary damage in experimental neonatal lung injury	Neonatal lung injury	hAECs	Preclinical	✓ Improving lung architecture. ✓ Reducing macrophages, DC, NK, and pro-inflammatory cytokines.	IM and AI effects. Regenerative property.	Zhu et al. [10]
Amnion epithelial cell-derived exosomes restrict lung injury and enhance endogenous lung repair	Lung injury	EXO-hAECs	Preclinical	✓ Improving tissue-to-airspace ratio and reducing lung inflammation and fibrosis. ✓ Stimulating bronchioalveolar stem cell.	IM, AF and AI effects. Regenerative properties.	Tan et al. [11]
Human amnion epithelial cells as a treatment for inflammation-induced fetal lung injury in sheep	Fetal lung injury	hAECs	Preclinical	✓ Attenuating lung function and structure. ✓ Reducing proinflammatory cytokines.	AI effect	Vosdogans et al. [12]
First-in-human administration of allogeneic amnion cells in premature infants with bronchopulmonary dysplasia: a safety study	Broncho- pulmonary dysplasia	hAECs	Clinical phase I	✓ Administration was safe and tolerated. ✓ No adverse events. (Completed)	-	Lim et al. [13]
Human amnion cells for the prevention of bronchopulmonary dysplasia: a protocol for a phase I dose escalation study	Broncho-pulmonary dysplasia	hAECs	Clinical phase I	✓ Safe and adverse events. (Recruiting)	-	Baker et al. [14]

*CM-hAMSCs* conditioned medium of human amniotic mesenchymal stromal cells, *EXO-hAMSCs* exosomes of human amniotic mesenchymal stromal cells, *hAMSCs* human amniotic mesenchymal stromal cells, *hAECs* human amniotic epithelial cells, *EXO-hAECs* exosomes of human amniotic epithelial cells, *DC* dendritic cells, *NK* natural killer cells, *CT score* computed tomography score

Effects: *AI* anti-inflammatory, *AF* anti-fibrotic, *IM* immunomodulatory

Zhang et al. [124] reported a potential hAMSC therapy for ARDS. They demonstrated that the transfection of Nrf2—a key transcription factor for antioxidant protein expression—in hAMSCs enhanced their protection effects in a murine model of lipopolysaccharide (LPS)-induced acute lung injury (ALI). Nrf2-transfected hAMSCs were able to ameliorate lung fibrosis and inflammation, improving the cell-based therapy for ALI and ARDS. Moreover, some authors have demonstrated that hAMSCs release soluble factors with regulatory actions over the injury site and exert trophic actions on host lung cells. In this way, Cargnoni et al. [49, 50] have shown that the hAMSC-CM reduced the fibrosis progression in a bleomycin-induced lung injury mouse model. They probed that the hAMSC-CM has the ability to protect the lungs from dysfunction caused by fibrotic lesions. In addition, they demonstrated that this CM reduced the profibrotic and inflammatory cytokine levels as well as T-lymphocyte and macrophage infiltration in the lungs. Notably, after comparing with CMs from other mesodermal origin cell types (PBMCs, Jurkat cells, human skin fibroblasts), they confirmed that the hAMSC-CM paracrine effects are specific to amniotic cells. These results support paracrine mechanisms of hAMSCs in their anti-inflammatory and anti-fibrotic effects.

## Human amniotic epithelial stem cells

Human amniotic epithelial cells have advantages over other stem cells for their use in cell therapy. HAECs derive from the pluripotent epiblast, which gives rise to the three germ layers of the embryo: endoderm, mesoderm, and ectoderm [125]; they express markers normally present in embryonic or germ cells and have several characteristics that make them attractive for regenerative medicine [126, 127]. Their differentiation potential has been studied by several research groups. In particular, pancreatic [127–130], pulmonary [131], hepatic [132–138], osteogenic [139], chondrogenic [140], adipogenic [139], neuronal [141, 142], and cardiac [127, 143] differentiation has been reported. Moreover, hAECs have low immunogenicity and anti-inflammatory properties, their isolation is simple, and their origin does not possess ethical concerns. They are obtained in great quantity, with around 100 million cells per amnion [104, 139, 144–147]. Also, they do not express telomerase and they are not tumorigenic after transplantation [127].

The clinical potential of hAECs is based essentially on their paracrine mechanisms that are capable of inducing anti-inflammatory and immunoregulatory responses [81]. HAECs express and secrete HLA-G that allows allograft acceptance and correlates with a low frequency of rejection [134]. Indeed, HLA-G modulates the immune response of Treg, myeloids, and NK cells, by binding to specific receptors in immune cells [148, 149]. In addition, hAECs express CD59 and the Fas ligand (FasL), involved in T lymphocyte complement activity inhibition and B lymphocyte apoptosis induction [150–152]. HAECs also secrete the migration inhibitor factor (MIF) that prevents neutrophil and macrophage migration ability [151].

Several studies have reported the ability of hAECs to reduce inflammatory cytokines and to release soluble anti-inflammatory factors [104, 145, 153]. Moreover, hAECs induce a Treg switch, altering the surrounding microenvironment by stopping inflammation [154, 155]. Additionally, hAECs express IDO [156], an enzyme involved not only in the cell cycle arrest and apoptosis of active CD4+ and CD8+ T cells, but also in Treg differentiation to resting CD4+ T cells [157]. Importantly, hAECs are capable to block the beginning of an immune reaction by changing the antigen-presenting function of DC. Amniotic cells impair the DC function and maturation in vitro [108]. Since the DC presented antigen will trigger an immunotolerance response, the manipulation of these cells is critical for stem cell therapy.

Indeed, hAECs can improve the inflammatory and fibrotic microenvironment that occurs in lung injuries. Moodley et al. [131] demonstrated the anti-inflammatory and anti-fibrogenic effect of hAECs when transplanted into a bleomycin-induced lung injury mouse model. Injection of hAECs reduced levels of proinflammatory cytokines such as IL-1β, IL-6, TNF-α, and TGF-β, a pulmonary fibrosis inductor. Furthermore, these authors demonstrated that hAECs differentiate into surfactant factors producing epithelial cells, both in vitro and in vivo conditions. While hAECs could adopt a type II pneumocyte phenotype, they may be able of longer-term alveolar restitution. However, we should consider the possibility of differentiated hAEC infection by SARS-CoV-2. This could be a limitation that still needs to be study. Also, this work together with a similar one [158] showed that hAECs promote collagen deposit degradation, since they induce an increase in metalloproteinases expression and an inhibition of tissue inhibitors of metalloproteinases. Besides, hAECs can release factors such as IFN-γ and MCP-1 that regulate the inflammatory macrophages migration to lung lesions. HAECs increase the levels of IL-10 [159, 160]. Another study also showed that hAEC administration modulated the pulmonary inflammatory response after ventilation and reduced lung injury in preterm lambs [161]. Additionally, early administration of hAECs improved the pulmonary architecture activating bronchioalveolar stem cell niche, in a neonatal lung injury murine model [162]. Moreover, hAECs have been able to modulate LPS-induced fetal pulmonary inflammation and to reduce proinflammatory cytokines levels in a sheep model [163].

On the other hand, hAEC-derived exosomes have been involved as modulators of the macrophage phenotype, inducing their differentiation from pro-inflammatory M1 type to anti-inflammatory M2 type [164].

In 2015, Lim et al. [165] reported the first hAEC phase I clinical trial, conducted in patients with chronic lung disease. Amniotic epithelial cells were administered intravenously at a dose of 1 million cells per kilogram body weight, in 6 premature babies with bronchopulmonary dysplasia (BPD). The patients were followed for 2 years. They concluded that hAEC administration is safe and does not cause adverse effects [165]. More recently, a phase I study was initiated in newborns with severe BPD. Since is unlikely that 1 million cells will be enough for a successful treatment, this study aims to evaluate the safety of higher doses of hAECs. The authors will assess the dose escalation after intravenously administration of up to 30 million hAECs per kilogram in 24 premature babies with BPD.

These results will be published in mid-2022, where the authors will include a cytokine profile description of patients after cell injection [166]. Also, other phase I clinical trials have begun to assess the safety of allogeneic hAEC transplantation in different pathologies such as ischemic stroke [167] and hepatic fibrosis [168]. These studies will provide evidence for subsequent trials. Preclinical and clinical trials related to lung disease treatment with hAECs are summarized in Table 3.

Up to this point, the reported benefits of hAEC transplantation in lung injury models make them a remarkable resource for lung regeneration. Since hAECs reduce inflammatory cell infiltration, fibrosis, and cytokine production and differentiate into alveolar epithelial cells, a COVID-19 treatment with hAECs needs to be considered.

## Non-cellular therapies for COVID-19

The ACE2 receptor or TMPRSS2 blocking in host cells could be one way for therapeutics elaboration against SARS-CoV2. Indeed, there are some clinically approved drugs that target those molecules for other treatments [169]. Antibodies against the spike protein (to block the interaction with ACE2), or protease inhibitors against TMPRSS2 (to prevent spike protein cleavage) may be other possible therapies. The use of virus memory CD8+ T cells could be effective to destroy infected cells. An alternative published treatment consists in the passage of infected patient blood though affinity columns to trap proinflammatory cytokines [170].

Other currently available clinical therapies include respiratory support (mechanical ventilation, non-invasive ventilation or invasive ventilation), non-specific antiviral drugs (remdesivir, lopinavir-ritonavir), plasma from recovered patients, and anti-inflammatory treatments such as NSAIDs, glucocorticoids (dexamethasone), aminoquinolines (chloroquine and hydroxychloroquine), immunosuppressants, and inflammatory cytokine antagonists [171].

Chloroquine and hydroxychloroquine are old drugs developed for the treatment of malaria. They are also used to treat systemic lupus erythematosus and rheumatoid arthritis. They are both 4-aminoquinolines [172]. Both drugs present immunomodulatory properties, prophylactic, and antiviral activity in vitro and have been shown to effectively act against the SARS-CoV-2 [173]. Thus, several clinical trials have been triggered with these drugs to treat and prevent COVID-19. It has been demonstrated that hydroxychloroquine has a more potential antiviral effect, so most studies are performed with this drug instead of chloroquine [174]. All the clinical trials performed until now made arise several doubts and questions regarding the use of chloroquine and hydroxychloroquine to treat COVID-19 patients. The studies were small and some of them report beneficial effects [175, 176] and some harmful [177]. Since all these reports had important limitations and they were small trials, questions and controversy around the use of hydroxychloroquine for COVID-19 treatment are still in the middle of the scene. Even the FDA that on March 28, 2020, had approved its emergency use, on June 15, 2020, cancelled this authorization due to the accumulation of negative data.

As dexamethasone is a glucocorticoid capable of modulating inflammation derived from COVID-19 lung injury, it has been specially considered as a drug treatment. On July 2020, the FDA included dexamethasone sodium phosphate to the list of drugs for temporary compounding by outsourcing facilities and pharmacy compounders, and it was kept on the list until now. Yet, the effectiveness of glucocorticoids against COVID-19 is still in doubt. The main study with dexamethasone involved the participation of 11500 patients from different UK hospitals [178]. Half of the patients received oral or intravenous dexamethasone. Comparison was made between those receiving dexamethasone and usual therapy and those receiving usual therapy alone. After 28 days of treatment, dexamethasone produced one third deaths reduction in mechanically ventilated patients and one fifth reduction in those with oxygen only. The mortality was the same in patients without any breathing help, treated or not with dexamethasone. Moreover, there was a chance of harm after dexamethasone treatment. Before this trial, glucocorticoids use for COVID-19 was contraindicated [179]. Thus, despite dexamethasone is now included in the recommended NIH and FDA drug list to treat COVID-19 hospitalized patients [178, 180], its use should be cautious and carefully considered in each case.

Remdesivir is an adenosine nucleotide analogue that acts as antiviral drug in a broad spectrum. Its emergency use has been approved by the FDA [181] as one of the few helpful molecules to treat COVID-19. Initially, remdesivir was developed to combat Ebola and now there are different clinical data reporting its therapeutic effect against the SARS-CoV-2. One of these trials (phase III) reported that the recovery time in hospitalized COVID-19 patients was shorter when remdesivir was used [182]. On the other hand, another study found no differences in clinical status between remdesivir and the standard treatment [183]. Anyway, remdesivir use was allowed by the FDA in adults and children with severe COVID-19, since its effect overcome the placebo in patients recovery time and improves general condition of the respiratory tract. Nevertheless, high COVID-19 mortality continues to occur; thus, combinations of therapeutics methods and drugs should be careful evaluated in the future to get better results.

Other antiviral that has been tested in some clinical trials is lopinavir-ritonavir, an HIV protease inhibitor. However, after negative pharmacodynamic data and no clear evidence of benefit, the NIH has recommended to avoid it use for COVID-19 patients [184].

Administration of convalescent plasma obtained from patients recovered from COVID-19 is a safe procedure that seems to have no adverse effects. The main issue is time for administration since it has to be injected as early as possible to achieve maximum efficiency, but the targets are severe cases [185]. However, so far all the evidence regarding convalescent plasma is positive and support its use [186].

Despite the scientific community efforts to mitigate the effects of this pandemic, treatments to control SARS-CoV-2 virus are not yet effective. There is an urgent need to find safe and effective treatments, particularly in severe cases.

## Perspectives on amniotic stem cells for COVID-19

Recently, COVID-19 has attracted the global scientific community full attention. The human and economic losses caused by the pandemic have accelerated the search for new effective and safe therapies against COVID-19. Stem cells, and especially human amniotic membrane stem cells, could be considered for future clinical research against SARS-CoV-2 virus. However, it is necessary to develop preclinical studies and clinical trials with proven efficacy, especially in coronavirus-induced respiratory diseases.

In this way, the paracrine activity of hAMSCs and hAECs could suppress the exacerbation of the immune system in ARDS caused by the SARS-CoV-2 virus. Amniotic membrane cells would allow a secretion decrease of pro-inflammatory cytokines at the lung injury focus and would attenuate the migration of neutrophils, macrophages, and lymphocytes. Removal of these inflammatory mediators would probably lead to a suppression of the cytokine storm and would prevent a multiple organ failure progression. Moreover, hAMSCs and hAECs could modulate the proliferation and activation of immune cells, inducing a switch to an anti-inflammatory phenotype.

Furthermore, amnion cells would not only promote the proinflammatory environment modulation but also a replacement of the diseased cells. In addition, human amniotic membrane cells could counteract fibrosis by decreasing factors for instance TGF-β [131].

Notably, the expression of HLA-G in hAMSCs and hAECs will allow a better transplant tolerance since this molecule acts suppressing NK cytotoxicity and modulating T cell and B cell proliferation and activity [187].

It is worthy to mention that amnion cells have a limited expansion capacity and they may lose their beneficial abilities through passages. Importantly, new protocol development would improve amniotic cell quantity and quality for clinical use. Some of them are in progress [138].

In summary, due to their differentiation capacity and its undoubted immunoregulatory properties, amnion cells therapy would be a promising treatment for COVID-19. They will probably act dually, not only by the prevention of cytokine storm but also by repairing and replacing damaged tissue. These features position human amniotic membrane stem cells as candidates to develop new successful protocols and curative treatments for COVID-19.

## Conclusions

Undoubtedly, the SARS-CoV-2 virus appearance together with the COVID-19 disease has changed the world as it was known until present. A problem of such dimensions commits the scientific community to develop quick and effective solutions, but mainly safe. It is urgent to find successful curative treatments. For this reason, we must first stop to analyze the pre-existing truthful information that allows us to hypothesize about the clinical effectiveness of possible therapies. Stem cells represent the great hope for regenerative medicine, and they stand out in the search of this information. In particular, due to their set of immunoregulatory, reparative, and regenerative properties, stem cells derived from the amnion of the human placenta should be considered for the treatment of this type of pulmonary affections as well as for a wide range of diseases.

## Data Availability

The original contributions presented in the study are included in the article/supplementary materials, and further inquiries can be directed to the corresponding author/s.

## Abbreviations

ACE2: Angiotensin-converting enzyme 2 receptor
ALI: Acute lung injury
ALT: Alanine aminotransferase
ARDS: Acute respiratory distress syndrome
ASC: Adipose stem cells
AST: Aspartate aminotransferase
BM-MSC: Bone marrow mesenchymal stem cells
BPD: Bronchopulmonary dysplasia
COVID-19: Coronavirus disease-2019
CoVs: Coronaviruses
CRP: C reactive protein
CT: Computed tomography
DC: Dendritic cells
FasL: Fas ligand
FDA: Food and Drug Administration
GGO: Ground-glass opacity
GSCF: Granulocyte colony-stimulating factor
GVHD: Acute refractory graft-vs-host-disease
hAECs: Human amniotic epithelial cells
hAMSCs: Human mesenchymal stromal cells
hAMSC-CM: Human mesenchymal stromal cells conditioned medium
HAT: Human airway trypsin-like protease
hESC: Human embryonic stem cells
HGF: Hepatocyte growth factor
HLA-G: Human leukocyte antigen G
hUCMSC: Human umbilical cord mesenchymal stem cells
IDO: Indoleamine 2,3-dioxygenase
IFN-γ: Interferon-gamma
IP-10: Interferon γ inducible protein
iPSCs: Induced pluripotent stem cells
LPS: Lipopolysaccharide
MCP1: Monocyte chemo-attractant protein 1
MIF: Migration inhibitor factor
MIP1A/B: Macrophage inflammatory protein 1 alpha/beta
MSC: Mesenchymal stem cells
NIH: National Institutes of Health
NSAIDs: Nonsteroidal anti-inflammatory drugs
NK: Natural killer cells
ORFs: Open reading frames
PBMC: Peripheral blood mononuclear cells
PGE2: Prostaglandin E2
SARS-CoV-2: Severe acute respiratory syndrome coronavirus
SVF: Stromal vascular fraction
TGF-β: Transforming growth factor-beta
TNF-α: Tumor necrosis factor-alpha
Treg: T regulatory cells
TSG-6: Tumor necrosis factor stimulated gene-6
VEs: Extracellular vesicles
VEGF: Vascular endothelial-derived growth factor
WHO: World Health Organization
